# Research Trends and Development Patterns in Language Testing Over the Past Three Decades: A Bibliometric Study

**DOI:** 10.3389/fpsyg.2022.801604

**Published:** 2022-02-09

**Authors:** Manxia Dong, Cenyu Gan, Yaqiu Zheng, Runsheng Yang

**Affiliations:** ^1^School of Business English, Sichuan International Studies University, Chongqing, China; ^2^Aoping Middle School, Chengdu, China; ^3^School of English Studies, Sichuan International Studies University, Chongqing, China

**Keywords:** language testing, bibliometric analysis, research trends, most frequently researched test types, most frequently discussed topics, most cited publications and authors, most prolific countries and institutes, most frequent collaborative countries/regions

## Abstract

This study used bibliometric data from *Language Testing, a* prestigious international peer-reviewed journal in the language testing field, to investigate research trends and development patterns in language testing. The bibliometric information included the number of publications, the most frequently researched test types and topics, the most cited publications and authors (as measured by references), the most prolific countries/regions and institutions and the most frequently collaborating countries/regions. The results showed that interest in language testing has increased over time and that regional tests and international tests have been major concerns, while classroom tests/assessments have received less attention. Research topics were wide-ranging and addressed almost all language testing related issues, among which validity/validation received the highest interest across periods. Moreover, the publications were produced by a wide range of countries/regions and institutions and included collaborative research spanning various institutions and regions, although collaborative publications across countries were relatively scarce. Based on the findings of this study, implications and suggestions have been highlighted for future research, academic agencies and this journal.

## Introduction

Language testing is frequently used as a criterion for measuring language abilities in second language acquisition research, and this measure can serve as a valuable information source for making decisions within the context of education and as an indicator in assessing abilities or attributes that are of interest in research on language, language teaching and language learning (Bachman, 1990). Bachman (1991) further found that although there are various uses of tests, these uses can be classified into two primary categories. The first category includes situations in which language testing results are used to make inferences or predictions about the language ability of the test-takers’ or their capacity to perform future tasks outside of the test context. The second category includes situations in which test results are used to make decisions about test-takers, such as selection, grading, and placement progress on the basis of inferences from test scores about test-takers’ levels of ability in language use in non-test situations. Due to its critical role in language teaching, learning, research and decision-making within the context of education, language testing has received wide attention from researchers and educators, and a multitude of studies on language tests have been conducted and published.

There are only a very small number of journals in the language testing/assessment field, such as *Language Testing*, *Language Assessment Quarterly*, and *Assessing Writing*. Among these few journals, *Language Testing*, as the most prestigious international peer-reviewed journal, “has become a venue publishing original research on foreign, second and bi-/multi-/*trans*-lingual language testing, assessment and evaluation” (Language Testing, 2021). Since it was established in 1984, the journal has had a great deal of impact and attained great popularity in the language testing/assessment field. Every academic journal has its own stated aims and scopes. Lei and Liu (2019a) claimed that it is of vital interest and importance for stakeholders (e.g., publishers, editors, and the editorial board), potential readers and contributing authors to know whether and how successful a journal has been in achieving its aims and staying within its scopes. This reason for their claim was that such information may help stakeholders to make informed decisions on the research issues to investigate, research funding allocation and language policy formulation and for professors to obtain a better understanding of research trends and hotspots (Lei and Liu, 2019b). Thus, this study attempts to use a bibliometric analysis of publications in the *Language Testing* journal over the past three decades (1984 to 2020) to provide such information as a means of revealing trends and development patterns in language testing research. These research results are also helpful in understanding whether the stated aims and scopes of the journal have been fulfilled and in identifying implications and suggestions for the journals’ stakeholders (e.g., publishers and editorial board), contributing authors and potential readers.

## Review of Bibliometric Research

The earliest bibliometric study dates back to the end of the 19th century, although the term ‘bibliometrics’ was coined fairly recently (Osareh, 1996). Numerous researchers and scholars have defined the term ‘bibliometrics’ (e.g., Pritchard, 1969; White and McCain, 1989). For instance, White and McCain (1989, p. 119) defined bibliometrics as “the quantitative study of literatures as they are reflected in bibliographies.” Early bibliometric studies were mostly restricted to fields in the natural sciences and concentrated on the knowledge development of a discipline rather than on the impact and productivity of research in that discipline (Lei and Liu, 2019b). With modern technological development, tremendous changes in bibliometric study methods have occurred, especially after the official release of the Science Citation Index (SCI) in 1963 (Gingras, 2016). Due to its unique citation method and comprehensive scientific data, including data concerning the citation frequency of a certain article and the impact factors of a journal, the SCI can provide a basis for making a reasonable judgment about the scientific research merits of a country, region, research unit or individual to reflect the international academic level of a specific target. Consequently, the SCI is currently recognized as the most authoritative scientific literature search tool in the world.

As a companion volume to the SCI, the Social Sciences Citation Index (SSCI) covers social science fields, such as anthropology, law, economics, history, geography and psychology. Using both the SCI and the SSCI, a wealth of bibliometric information can be searched and retrieved more easily from datasets, including authors, institutions, countries, citations of publications, and collaborative publications. Such information is useful for evaluating the contributions of authors, institutions and countries/regions to a field or a journal, as well as the impacts of researchers, articles and journals (De Bellis, 2009; Leydesdorff and Wagner, 2009). Lei and Liu (2019b) proposed that bibliometric analysis is especially effective for identifying major research topics and trends in a field. However, some scholars have suggested that caution should be exercised when using bibliometric data to assess researchers, institutions and countries because these data might be misinterpreted, misused or abused due to the potential for misunderstanding (van Raan, 2005; Gingras, 2016; Lei and Liu, 2019a).

There have been numerous bibliometric studies on general and specific disciplines in the natural and social sciences in the recent decades (e.g., Leydesdorff and Wagner, 2009; Moiwo and Tao, 2013; Liu et al., 2015). However, there have been only a few such studies on linguistics and applied linguistics (e.g., van Doorslaer and Gambier, 2015; Liao and Lei, 2017; Lei and Liu, 2019a,b). Some of bibliometric studies examined the research throughout the entire discipline of linguistics/applied linguistics (e.g., Lei and Liu, 2019b) and publications in certain subareas, such as second or foreign language teaching (e.g., Gong et al., 2018) or corpus linguistics (e.g., Liao and Lei, 2017), while others focused on an individual journal to examine whether its mission had been achieved and to uncover research trends and development patterns. For instance, Lei and Liu (2019a) used article data from 42 journals indexed in the Social Science Citation Index (SSCI) to reveal research trends and hot topics throughout the entire field of applied linguistics. Their research provided a general and overall understanding of research trends in the applied linguistics field. Lei and Liu (2019a) focused on an individual journal “*System*” in the applied linguistics field to investigate research themes and evolving patterns using bibliometric data concerning *System* over four decades. Based on their research results, Lei and Liu (2019a) offered suggestions for adjustments or improvements that the journal could make to achieve its declared aims and scopes and provided useful information to help readers and potential contributors confirm the research foci of this journal and to aid them in targeting their studies toward the journal.

In the language testing/assessment field, only a very small number of bibliometric studies have been performed (e.g., Jiang, 2018; Zhang et al., 2021). Jiang (2018) conducted a bibliometric study on the foreign language testing field based on retrieved data published in 14 foreign language journals in China from 2006 to 2017. However, her research did not reflect international research trends in the language testing/assessment field because her study only included domestic journals and did not involve international journals. Zhang et al. (2021) retrieved data from 10 international journals from 2008 to 2018 that published language testing/assessment papers via the *Web of Science Core Collection*. Their study focused on bibliometric information, including the most productive countries, regions and authors, high-impact publications and authors and research hotspots. Their study provided a general picture of the research trend but did not address the development of and changes in language testing/assessment over time. Given the large difference in the number of publications published each year by each journal and in the number of years each journal listed in the SSCI index has been in operation, it is difficult to conduct valid and meaningful comparisons among journals. Thus, we decided to conduct a bibliometric study on an individual journal in the language testing/assessment field. *Language Testing*, as the earliest, highest impact and most popular journal, better reflects the research trends in and the development of language testing. Thus, this study targeted *Language Testing* to conduct a bibliometric analysis for the purposes of uncovering information regarding the journal for professionals and organizations in this field as well as providing guidance for future research. Through an analysis of 759 publications from 1984 to 2020 in *Language Testing*, the following six research questions were addressed:

(1) How many publications did *Language Testing* produce per year and period?
(2) What types of tests were most frequently researched in the journal?
(3) What topics received the most attention from researchers?
(4) Which publications and authors (as measured by references) had the most citations?
(5) Which countries/regions and institutions were the most prolific in terms of research production?
(6) Which countries/regions were the most prolific in producing collaborative publications?

## Materials and Methods

### Dataset and Data Search

A total of 759 bibliometric items published in *Language Testing* between 1984 and 2020 were downloaded from the Scopus database. In this study, we used Scopus to retrieve data instead of Web of Science because *Language Testing* was not included in the Web of Science database until 2008 but was listed in Scopus beginning in 1984, when the journal was established.

After retrieval and a preliminary analysis of the original data, five document types amounting to a total of 805 publications published in *Language Testing* from 1984 to 2020 were collected, including articles (*N* = 723, 89.8%), reviews (*N* = 36, 4.5%), editorials (*N* = 40, 5.0%), errata (*N* = 5,0.6%), and notes (*N* = 1, 0.1%). Data for the years 1984 to 2020 were downloaded from Scopus on December 13, 2020. In this study, only articles and reviews, amounting to a total of 759 documents, were involved in the data analysis.

### Data Analysis

We analyzed and reported the results for the entire period (1984–2020) to produce an overall bibliometric picture for this journal. In addition, we referred to Lei and Liu (2019a,b) and divided the entire 37-year time period into subdivision periods to ascertain differences or changes across periods. In this study, the 37 years were divided into three subperiods instead of five calendar decades for analysis mainly because the 1990s, 2000s, and 2010s each include one full decade, while the 1980s include only 6 years and the 2020s include only 1 year. Our rationale for the division into three periods (1984–1995, 1996–2007, and 2008–2020) was twofold. First, we hoped to have a relatively even split of the 37 years of publication into three periods, which means that two periods included 12 years, while one period included 13 years. Second, *Language Testing* has received wider attention from researchers throughout the world since its bibliometric information was included in the Web of Science in 2008, which marked a new period of language testing research. Thus, we allocated the retrieved publications that appeared from 2008 to 2020 into the third period, and then the retrieved publications from 1984 to 2007 were evenly divided into two periods. By searching and analyzing the data from 1984 to 2020, we obtained the following information, which allowed us to respond to the six research questions stated above.

The bibliometric information concerning the number of publications, most cited publications and authors (as measured by references) (Top 10), most prolific countries/regions and institutions (Top 10) for the entire 37 years and for each time period was directly retrieved from the Scopus database to address the first, fourth and fifth research questions. However, the bibliometric information concerning the most frequently researched test types, most frequently explored research topics and most frequently collaborative countries/regions could not be directly retrieved from Scopus data. To address those three questions, we used Microsoft Excel 2010 and manual analysis to collect and analyze the data. Specifically, the data were first downloaded from the Scopus database and entered into Microsoft Excel 2010, including the research title, abstract, year, author, contributing country and contributing institution. The bibliometric information was then analyzed manually, and the data analysis results were recorded and computed in Microsoft Excel 2010. The detailed data collection and analysis used to address research questions 2, 3, and 6 are illustrated as follows.

The second research question aimed to identify the most frequently researched test types in *Language Testing*. *Language Testing* is a journal that publishes original research on foreign, second and bi-/multi-/*trans*-lingual language testing and assessment around the world. Thus, the types of language tests that have been researched may be of much interest to readers and researchers. Regarding the classification of test types, there are various criteria. Jiang (2018) analyzed the tests using scales, including large-scale tests and school-based tests. Through preliminary analysis of the research titles and abstracts of all publications and based on Jiang’s (2018) classifications on test types, the tests were analyzed in terms of the range/scale dimension, including international language tests, regional language tests and classroom language tests/assessments. In this study, international language tests referred to large-scale, high-stakes international language tests, e.g., the International English Language Testing System (IELTS) and the Test of English as a Foreign Language (TOEFL). Regional language tests represented local tests developed and administered within a country or region, including national criterion/norm-referenced tests and school-based tests, i.e., the Test for English Majors (TEM) in China. Classroom language tests/assessments referred to tests/quizzes or assessments administered in the classroom. To ensure reliability of the analysis, three researchers conducted a joint analysis of the title and abstract of each publication item, and divergences in the analysis were addressed to obtain agreement through discussion or were submitted to a language testing expert for final verification. Notably, some publications that cannot be definitively classified into the above test types were excluded from the data analysis. For instance, some research explored tests in terms of test formats, e.g., cloze or writing tests; consequently, we could not identify the exact test types from these publications. Ultimately, only 350 publication items were identified and included in the final analysis.

Regarding the bibliometric data on the most frequently explored topics (RQ3), a review of the literature revealed that in previous studies, research topics or themes were usually identified through keyword analysis using AntConc software (e.g., Lei and Liu, 2019a,b), through clustering analysis using CiteSpace, a visualization tool in scientometrics (e.g., Kong, 2017; Zhang et al., 2021) or through theme analysis conducted via manual analysis and verification of publications by multiple researchers (e.g., Zou and Dong, 2014; Dong et al., 2021). However, we found that before 2008, *Language Testing* did not provide key words for publications. Thus, identifying research topics by analyzing key words was impractical in this study. Clustering analysis of all the publications using CiteSpace may be time-saving and allow for high reliability in identifying research topics or themes. However, we also found that the results of clustering analysis of all the publications using CiteSpace did not provide meaningful research topic descriptions in language testing. Although clustering analysis of key words can better reflect research topics, this approach is still not applicable because key words were unavailable for the journal before 2008. Thus, our study attempted to adopt theme analysis to identify topics. The title and abstract of each publication reflect the research focus and aims of the publication to some extent. Three researchers first read the titles and articles, and then identified major research topics in language testing research, such as validity, reliability, impact, authenticity, scales and test ethics, through repeated discussion. Given that reviews were not directly related to the research topic, they were excluded from the topic analysis. Finally, only article publications (*N* = 723) were involved in the data analysis. The same data analysis steps as those used to address the second research question were used to advance the analysis reliability. During the analysis, some publication items that failed to be included in the list of major topics were identified as new research topics. According to the analysis results for the research topics, the top 10 most frequent research topics were identified using Microsoft Excel 10.

To address the question of the countries/regions that engage in collaboration most frequently (RQ 6), we extracted bibliometric information from the downloaded data and computed the frequencies and percentages of collaborative publications, international collaborative publications and the number of collaborative countries to identify the top 10 countries/regions that engage in collaboration most frequently.

## Results and Discussion

### The Number of Publications Per Year and Per Period

**FIGURE 1:**
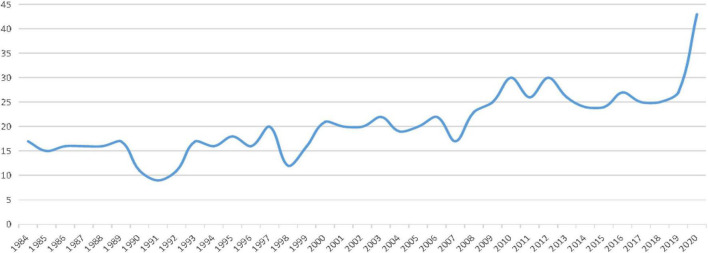
Publications published in *Language Testing* from 1984 to 2020.

The distribution of publications in *Language Testin*g during the 1984–2020 period is presented in Figure 1. The results illustrated that although the annual number of research publications fluctuated slightly, a steady increase is observed overall. The years from 1990 to 1992 exhibited the smallest number of publications, and the publication rate has been increasing since 1992. The year 1990 was considered to be a watershed moment in the development of language testing (Douglas, 1995) because in that year, several major events in the field of language testing occurred. For instance, the *12th International Language Testing Seminar* was held in San Francisco, United States, and a seminar on *Language Testing and Project Evaluation* was held in Singapore. In addition, certain important academic language testing treatises appeared in the same year, e.g., *Fundamental Consideration in Language Testing* by Bachman (1990) and *Principles of Language Testing* by Davies (1990), which played a crucial role in promoting language testing development (Yang, 2002).

The publication quantities over the past three decades and for each period are presented in Table 1. The results showed that the annual average number of publications over the three decades was 20.5, with a minimum value of 9 and a maximum value of 43. Further analysis showed that the publication rates increased over time. Specifically, in the period 1984–1995, 179 publications (23.6%) were published, with an average annual number of publications of 14.9 and with minimum and maximum numbers of publications of 9 and 18, respectively. The number of publications during this period fluctuated but continued to increase slightly. In the period 1996–2006, 225 publications (29.6%) were published, with an average of 18.8 publications per year and a minimum and maximum of 16 and 22 publications per year, respectively. The number of publications published in this period fluctuated slightly but remained steady. In the period 2008–2020, 355 publications (46.8%) were published, with an average of 27.3 publications per year and a minimum and maximum of 23 and 43 publications per year, respectively. The annual number of publications during this period increased rapidly, and the peak number of publications reached 43 in 2020.

**TABLE 1:** Publication quantity stratified by period.

Bibliometric indicators	1984–2020	1984–1995	1996–2007	2008–2020
Number of publications (%)	759 (100%)	179 (23.6%)	225 (29.6%)	355 (46.8%)
Average annual publication	20.5	14.9	18.8	27.3
Minimum/maximum	9/43	9/18	16/22	23/43

### The Most Frequently Researched Test Types

Table 2 shows that the journal included research related to international language tests, regional language tests and classroom language tests/assessments. Among the three types of tests, regional tests received the most attention (*N* = 184, 52.6%), followed by international tests (*N* = 120, 34.3%). Compared with international language tests and regional language tests, classroom language tests/assessments received the least attention (*N* = 46, 13.1%). A plausible explanation for this result is that international language tests and regional language tests, as large-scale, high-stakes tests, are more likely to receive attention from researchers due to their great influence on teaching, learning and even society at large.

**TABLE 2:** Most frequently researched test types.

	1984–2020		1984–1995		1996–2007		2008–2020	
	Publication (number)	%	Publication (number)	%	Publication (number)	%	Publication (number)	%
International tests	120	34.3	19	30.2	18	17.0	83	45.9
Regional tests	184	52.6	34	54.0	62	58.5	88	48.6
Classroom tests/assessments	46	13.1	10	15.9	26	24.5	10	5.5
Total	350	100	63	100	106	100	181	100

The comparisons among the three types of tests showed that regional language tests accounted for the largest percentage of publications during each period (approximately 50%), suggesting that regional language tests received the most attention across the three periods. This result was probably because many countries or regions have developed L2 or foreign language tests. These regional L2 or foreign language tests are normally large-scale, high stakes tests; thus, they more easily attract the attention of researchers. It is no surprise that international language tests, such as large-scale high-stakes tests, have also received a great deal of attention from researchers due to their great influence worldwide. Notably, during the third period, the research on international language tests greatly increased to 45.9%, while research on classroom language tests/assessments sharply declined to 5.5%. The results indicated that international language tests have become increasingly popular, while less attention has been given to classroom language tests/assessments. This result probably stems from the development of new international language tests or the implementation of international language test reforms in the recent period. The result showed greater concern from researchers about international language tests, and naturally, less attention was given to classroom language tests/assessments.

### The Most Frequently Discussed Research Topics

By using the above-described procedures to analyze the titles and abstracts of 723 article publication items, we identified the 10 most frequently discussed research topics that were identified over the past three decades (see Table 3). The top 10 topics were the subject of 478 publications, accounting for 66.1% of total article publications, and included the topics of validity/validation, reliability, test design and development, theory and framework, test items, individual characteristics, scale, vocabulary size/richness/diversity, test impacts and test ethics. Validity and reliability were defined as two essential qualities of interpreting and using language ability measures (Bachman, 1990). In this study, we also classified these topics as two independent themes for a fuller and more specific description of research topics rather than understanding reliability as a part of validity based on the concept of unitary validity. The theory and framework topic refers to theoretical issues that have been defined as one of the primary research goals of *Language Testing*. This topic reflects the exploration and development of language testing theory. The test item topic represents relevant research concerning test items, e.g., differential item functioning (DIF). The individual characteristics topic represents research topics concerned with individual characteristics (e.g., test-takers and raters) in test development, administration and rating. Scale was listed as a single category because of its significance in language testing and its wide research scopes from the context of one specific exam to that of a large-scale language ability scale. Test ethics refers to research topics reflecting ethics concerns in developing and administrating a test, e.g., test fairness.

**TABLE 3:** Top 10 most frequently discussed research topics.

Order	1984–2020		1984–1995		1996–2007		2008–2020	
	Topics	Number (%)	Topics	Number (%)	Topics	Number (%)	Topics	Number (%)
1st	Validity/valuation	116(16.0)	Validity/validation	23(13.2)	Validity/validation	28(12.8)	Reliability	68(20.6)
2nd	Reliability	91(12.6)	Test design and development	23(13.2)	Test design and development	22(10.0)	Validity/validation	65(19.6)
3rd	Test design and development	77(10.7)	Theory and framework	9(5.2)	Reliability	15(6.9)	Test design and development	33(10.0)
4th	Theory and framework	48(6.6)	Test item	9(5.2)	Test item	13(5.9)	Theory and framework	32(9.7)
5th	Test item	42(5.8)	Reliability	8(4.6)	Scale	9(4.1)	Scale	22(6.7)
6th	Scale	28(3.9)	Individual characteristics	7(4.0)	Vocabulary size/richness/diversity	9(4.1)	Test item	20(6.1)
7th	Individual characteristics	25(3.5)	Scale	7(4.0)	Test ethics	9(4.1)	Individual characteristics	10(3.0)
8th	Vocabulary size/richness/diversity	19(2.6)	Test ethics	6(3.4)	Individual characteristics	8(3.7)	Vocabulary size/richness/diversity	10(3.0)
9th	Test impact	17(2.4)	Assessment method	4(2.3)	Theory and framework	8(3.7)	Test-takers’ performance	10(3.0)
10th	Test ethics	15(2.1)	Test-takers’ affective factors	4(2.3)	Test impact	6(2.7)	Test-takers’ affective factors	7(2.1)

Table 3 shows that validity/validation accounted for the largest proportion (*N* = 116, 16%), suggesting that validity/validation has remained a topic of highest interest in the language testing field, which resonates with the findings in Zhang et al.’s (2021) study. Kunnan (1998) commented that research on language testing and assessment has been concerned with validity since the 1960s. Reliability has been another important research topic in the language testing field, which was supported by the results of this study. In our study, the topic of reliability maintained a high level of interest, with a total of 91 publications, focusing on reliability, second only to the topic of validity/validation.

The results also revealed that some major research themes remained steady throughout the three periods. For instance, six highly prominent topics, including validity/validation, reliability, test design and development, theory and framework, test items, individual characteristics and scale, have gained great popularity in recent decades. The topic of reliability exhibited a constant increase, ascending from 4.6% during the period 1984–1995 to 6.9% in the period 1996–2007, and then climbing to 20.6% in the period 2008–2020, which suggests that research on reliability has received increasing attention in the recent decade.

In contrast to the popular topics, a few topics saw a distinct decrease in interest due to the development of language testing or shifts in research interest. For instance, the topic of test methods was highly popular during the early period of language testing research, but it has fallen out of fashion in recent periods due to the progress of technology and the development of language testing research. Similarly, test ethics was more frequently explored during the first and second periods but did not appear in the ranked list for the third period. The decline of test ethics research is perhaps easy to understand since the significance of test ethics has been widely recognized and since major considerations are applied by test developers and test-related authorities when designing and administrating tests. Therefore, researchers’ attention to the topic of test ethics has declined.

### The Most Cited Publications and Authors (As Measured by References)

#### The Most Cited Publications

The top 10 most cited publications between 1984 and 2020 are displayed in Table 4. From the table, we found that the number of citations of these publications ranged from 132 to 401. Among the articles, the most frequently cited publication with 401 citations was “*Developing and exploring the behavior of two new versions of the Vocabulary Levels Test”* by Schmitt et al. (2001, pp. 55–88). This publication was followed in the list of most publications by an article with 269 citations, “*Validity and washback in language testing”* by Messick (1996, pp. 241–256). The most highly cited recent publication also helped us identify the most popular publications and topics. The results showed that 5 of the 10 most cited publications were vocabulary-related research, and 3 of those publications were rater-related (defined as a part of reliability research in this study), indicating that vocabulary tests and raters have been the most popular themes during the past three decades. These results partly corroborate the findings above that vocabulary tests and reliability are the two most frequently explored research topics.

**TABLE 4:** Top 10 most highly cited publications.

Order	1984–2020		1984–1995		1996–2007		2008–2020	
	Title (author/year)	Number of citation	Title (author/year)	Number of citation	Title (author/year)	Number of citation	Title (author/year)	Number of citation
1st	Developing and exploring the behavior of two new versions of the Vocabulary Levels Test (Schmitt et al., 2001)	401	The development of a new measure of L2 vocabulary knowledge (Read, 1993)	174	Developing and exploring the behavior of two new versions of the Vocabulary Levels Test (Schmitt et al., 2001)	401	Pair versus individual writing: Effects on fluency, complexity and accuracy (Wigglesworth and Storch, 2009)	124
2nd	Validity and washback in language testing (Messick, 1996)	269	An alternative to multiple choice vocabulary tests (Meara and Buxton, 1987)	158	Validity and washback in language testing (Messick, 1996)	269	Rater types in writing performance assessments: A classification approach to rater variability (Eckes, 2008)	102
3rd	A vocabulary-size test of controlled productive ability (Laufer and Nation, 1999)	266	Rater characteristics and rater bias: Implications for training (Lumley and Mcnamara, 1995)	142	A vocabulary-size test of controlled productive ability (Laufer and Nation, 1999)	266	A Rasch-based validation of the vocabulary size test (Beglar, 2010)	87
4th	The development of a new measure of L2 vocabulary knowledge (Read, 1993)	174	Expertise in evaluating second language compositions (Cumming, 1990)	128	Using FACETS to model rater training effects (Weigle, 1998)	162	The key to success: English language testing in China (Cheng, 2008)	81
5th	Using FACETS to model rater training effects (Weigle, 1998)	162	Examining washback: The Sri Lankan Impact Study (Wall and Alderson, 1993)	107	Self-assessment in second language testing: A meta-analysis and analysis of experiential factors (Ross, 1998)	153	What makes speech sound fluent? The contributions of pauses, speed and repairs (Bosker et al., 2013)	75
6th	An alternative to multiple choice vocabulary tests (Meara and Buxton, 1987)	158	The effect of rater variables in the development of an occupation-specific language performance test (Brown, 1995)	106	Investigating accommodation in language proficiency interviews using a new measure of lexical diversity (Malvern and Richards, 2002)	138	Constructing a language assessment knowledge base: a focus on language assessment courses (Inbar-Lourie, 2008)	69
7th	Self-assessment in second language testing: A meta-analysis and analysis of experiential factors (Ross, 1998)	153	Effects of training on raters of ESL compositions (Weigle, 1994)	102	Assessment criteria in a large-scale writing test: what do they really mean to the raters? (Lumley, 2002)	132	Self-, peer-, and teacher-assessments in Japanese university EFL writing classrooms (Matsuno, 2009)	68
8th	Rater characteristics and rater bias: Implications for training (Lumley and Mcnamara, 1995)	141	Investigating variability in tasks and rater judgments in a performance test of foreign language speaking (Bachman et al., 1995)	89	Vocd: a theoretical and empirical evaluation (McCarthy and Jarvis, 2007)	128	Diagnostic assessment of writing: a comparison of two rating scales (Knoch, 2009)	67
9th	Investigating accommodation in language proficiency interviews using a new measure of lexical diversity (Malvern and Richards, 2002)	138	On taking language tests: what the students report (Cohen, 1984)	87	Interviewer variation and the co-construction of speaking proficiency (Brown, 2003)	123	The impact of EFL testing on EFL education in Korea (Choi, 2008)	67
10th	Assessment criteria in a large-scale writing test: what do they really mean to the raters? (Lumley, 2002)	132	Does the testing method make a difference? The case of reading comprehension (Shohamy, 1984)	82	A closer look at the relationship of cognitive and metacognitive strategy use to EFL reading achievement test performance (Phakiti, 2003)	122	The effects of self-assessment among young learners of English (Butler and Lee, 2010)	64

Notably, we found that in the top ten most cited publications, only two publications appeared after 2000, and the most recent publication year throughout the three decades was 2002. In the third period, the most recent publication year was 2013, while there were no publications from 2014 to 2020 in the rank list. This fact is partly because generally, a newer publication received fewer citations than an older publication when other variables remained constant (Lei and Liu, 2019a). This finding also relates to the age effect on the number of citations.

Further reading of the results showed that in the most highly cited publications vocabulary tests, rater, and test impact were the dominant themes throughout the three periods, which further confirmed the finding regarding the most frequently explored research topics. The results also revealed a shift in research focus across the three periods. During the first period, the articles pertained to vocabulary tests, raters, test impact, test method, and SL composition evaluation. During the second period, apart from the common dominant themes, writing, speaking and reading tests were explored. During the third period, the themes of assessment and writing received the most attention, where 5 of the 10 most highly cited articles focused on assessment, and 4 out of these 10 articles focused on writing. These findings showed that the journal *Language Testing* has maintained its focus on traditional testing-related issues while keeping pace with recent developments in the field.

#### The Most Cited Authors (As Measured by References)

By sorting all the authors in terms of references, we identified information regarding the 10 most cited authors (see Table 5). The results showed that among the top 10 most cited authors (as measured by references), Bachman was the author with the most citations (*N* = 716 times) over the past 37 years, followed by Alderson (*N* = 538) and McNamara (*N* = 453 times). Their classic books or papers make them the most highly cited authors throughout the whole period. For instance, regarding Bachman’s works, the top 3 highly cited publications in references are “*Fundamental considerations in language testing*” (Bachman, 1990), “*Basic concerns in test validation”* (Palmer and Bachman, 1981, pp. 135–151) and “*The construct validation of some components of communicative proficiency”* (Bachman and Palmer, 1982, pp. 449–465). However, an interesting finding is that several of the most cited authors listed in the top 10 (as measured by references) did not contribute any publications to the most cited publications ranking, such as Elder (7th), Oller (9th), and Henning (10th). Additionally, some authors with publications appearing in the most cited publications ranking are not included in the list of most cited authors. Through careful analysis of the results, we identified the following explanation for these facts: some authors had several of the most cited publications, which led to their high number of total citations, but they had no single seminal work with an extremely high number of citations, while other authors with one or two of the most cited publications scarcely made the rank list because of their low total number of citations.

**TABLE 5:** Top 10 most highly-cited authors (as measured by references).

Order	1984–2020		1984–1995		1996–2007		2008–2020	
	Author	Number	Author	Number	Author	Number	Author	Number
1st	Bachman, L.F.	716	Alderson, J.C.	100	Bachman, L.F.	318	Bachman, L.F.	305
2nd	Alderson, J.C.	538	Bachman, L.F.	93	Alderson, J.C.	240	McNamara, T.	264
3rd	McNamara, T.	453	Oller, J.W., Jr.	89	McNamara, T.	141	Alderson, J.C.	198
4th	Shohamy, E.	258	Klein-Braley, C.	85	Shohamy, E	120	Brown, A.	149
5th	Brown, A.	234	Henning, G.	84	Brindley, G.	96	Elder, C	144
6th	Brown, J.D.	207	Cohen, A.D.	61	Brown, J.D.	93	Fulcher, G.	133
7th	Elder, C	201	Spolsky, B.	54	Clapham, C.	92	Weir, C.	126
8th	Messick, S.	191	Hughes, A.	50	Messick, S.	89	Palmer, A.	119
9th	Oller, J. W., jr.	182	Carroll, J.B.	49	Palmer, A.	89	Chapelle, C.A.	95
10th	Henning, G.	177	McNamara, T.	48	Oller, J.W., jr.	85	Shohamy, E.	91

A closer examination of the results revealed that only Bachman, Alderson, and McNamara were listed among the top highly cited authors throughout the three periods. Among them, Bachman and Alderson remained relatively steady in rank (remaining in Top 3), and McNamara experienced a steady increase in the number of publication citations, from 48 times (rank 10) during the period 1984–1995, and 141 times (rank 3) in the period 1996–2007 to 264 times (rank 2) in the period 2008–2020. The results indicated that the seminal works of these authors significantly influenced the language testing field throughout the periods and have continued to receive attention from researchers. We also found that certain highly cited authors who emerged in the list/lists for one or two periods experienced a steady decrease in the number of citations, such as Cohen, Spolsky, Hughes, Carroll, Henning, and Oller. The results indicated that the publications of these authors received strong attention in one period or two periods, but that researchers’ interests or foci shifted as language testing developed.

### The Most Prolific Countries/Regions and Institutions

#### The Most Prolific Countries/Regions

The top 10 contributing countries are displayed in Table 6. Given that the number of publications during each period differed substantially, it is not valid or meaningful to simply compare the number of publications across three periods when analyzing the cross-period difference. Thus, we decided to compare the percentage of publications contributed by these countries/regions during each period rather than comparing the number. The results showed that over the past three decades, the top 10 contributing countries were responsible for 97.9% of the publications in the journal. Clearly, the United States was ranked first with the greatest number of scientific publications (*N* = 313), accounting for 41.2% of the total number of publications. Unsurprisingly, the United States led the world in research production. However, we found that Japan, Australia, Netherlands, China, South Korea, and Germany, also played leading roles in research production. A likely explanation for this result is that testing plays crucial roles in the education systems of East Asian countries, particularly in Japan, South Korea and China, which has been supported by some studies (e.g., Qi, 2004; Allen, 2016). Qi (2007) even claimed that the Chinese education system is characterized as a test-oriented system. Among these countries, the Netherlands and Germany, as bi-/multi-lingual language countries, have plentiful language tests, which may contribute to their higher publications. Australia’s high productivity was primarily due to the contribution from the Language Testing Research Centre (LTRC) of the University of Melbourne. This explanation was corroborated by the finding regarding the most prolific institutions.

**TABLE 6:** Top 10 most productive countries/regions.

Order	1984–2020			1984–1995			1996–2007			2008–2020		
	Countries/regions	Number	%	Countries/regions	Number	%	Countries/regions	Number	%	Countries/regions	Number	%
1st	United States	309	40.7	United States	81	45.2	United States	70	31.4	United States	158	46.3
2nd	United Kingdom	99	13.0	Israel	25	14.0	United Kingdom	45	20.0	Australia	40	11.7
3rd	Australia	83	10.9	United Kingdom	22	12.3	Australia	29	12.9	United Kingdom	32	9.4
4th	Canada	46	6.1	Australia	14	7.8	Canada	14	6.3	Canada	27	7.9
5th	China	40	5.3	Germany	7	3.9	China	12	5.3	China	25	7.3
6th	Japan	37	4.9	Netherlands	6	3.4	Netherlands	11	4.9	Japan	25	7.3
7th	Israel	35	4.6	Canada	5	2.8	Japan	10	4.4	Netherlands	14	4.1
8th	Netherlands	31	4.1	China	3	1.7	South Korea	9	4.0	South Korea	11	3.2
9th	South Korea	21	2.8	Iran	2	1.1	Israel	8	3.6	Germany	7	2.1
10th	Germany	21	2.8	Japan	2	1.1	New Zealand	8	3.6	Turkey	7	2.1

Table 6 shows that the United States, the United Kingdom, China, Australia, Netherlands, Japan, and Canada maintained their positions on the top ten rank lists throughout the three periods. Among these countries, Canada, China, and Japan continued to rise in rank order, and the other four countries remained relatively steady. However, Israel and Germany demonstrated a sharp fluctuation in terms of their rank order. For instance, Israel decreased from being ranked 2nd in the period 1984–1995 to being ranked 9th in the period 1996–2007 and finally did not make the list in the period 2008–2020. Germany was ranked 5th during the first period, did not make the rank list during the second period, and then reappeared on the list during the third period.

A closer examination showed that although most top contributors were Western countries/regions, e.g., the United States, the United Kingdom, and Australia, their number of publications actually decreased. Conversely, certain non-Western countries (i.e., China and Iran) exhibited a large increase in the number of publications. For instance, the percentage of publications produced in China increased from 1.7% during the period 1984–1995 and 5.3% in the period 1996–2007 to 7.3% in the period 2008–2020. The large increase in publications in non-Western countries indicates that the journal is aiming to accomplish its mission of becoming a venue for researchers in the language testing field worldwide, including developed and developing countries.

#### The Most Prolific Institutions

The top 10 contributing institutions over the past three decades are presented in Table 7. The results show that of the top 10 institutions, Educational Testing Service (ETS) was ranked 1st in institutional productivity, with 66 publications (8.8%). ETS, as an authoritative American institution with diverse and professional team members (e.g., researchers, statisticians, psychometricians, test developers, and education policy specialists), broad global markets and a wealth of test products and good test services, made a great contribution to *Language Testing and* played a leading role in the field of language testing. The second most productive institute was the University of Melbourne, contributing 57 publications (7.3%) to the journal. The University of Melbourne’s high productivity is primarily a result of the *Language Testing Research Centre* (LTRC), which was established in 1990. The central work of the LTRC “focuses around research and validation of language tests, test development, consultancies and industry linkages and has become an international leader in research and development in language assessment and language program evaluation” (Language Testing Research Center, 2021).

**TABLE 7:** Top 10 most productive institutions.

Order	1984–2020			1984–1995			1996–2007			2008–2020		
	Institution	*N* (%)	Country	Institution	*N* (%)	Country	Institution	*N* (%)	Country	Institution	*N* (%)	Country
1st	Educational Testing Service	66 (8.8)	United States	University of California, Los Angeles	15 (8.4)	United States	The University of Melbourne	16 (7.1)	Australia	Educational Testing Service	35 (9.9)	United States
2nd	The University of Melbourne	57(7.3)	Australia	Educational Testing Service	15 (8.4)	United States	Educational Testing Service	13 (5.8)	United States	The University of Melbourne	27 (7.6)	Australia
3rd	University of California, Los Angeles	34 (4.7)	United States	The University of Melbourne	11 (6.1)	Australia	University of California, Los Angeles	10 (4.4)	United States	University of Illinois system	13 (3.7)	United States
4th	Lancaster University	27 (3.4)	United Kingdom	University of Reading	8 (4.5)	United Kingdom	Lancaster University	9 (4.0)	United Kingdom	University of California system	12 (3.4)	United States
5th	University of Illinois at Urbana-Champaign	20 (2.1)	United States	Lancaster University	7 (3.9)	United Kingdom	University of Bristol	7 (3.1)	United Kingdom	University of Illinois at Urbana-Champaign	12 (3.4)	United States
6th	University of Hawai‘i at Mānoa	18 (1.9)	United States	University of Hawai‘i at Mānoa	6 (3.4)	United States	The Hong Kong Polytechnic University	6 (2.7)	China	Lancaster University	10 (2.8)	United Kingdom
7th	Iowa State University	14 (1.9)	United States	Tel Aviv University	6 (3.4)	Israel	The University of Auckland	5 (2.2)	New Zealand	University system of Georgia	10 (2.8)	United States
8th	The University of Edinburgh	13 (1.8)	United Kingdom	Hebrew University of Jerusalem	6 (3.4)	United States	University of Surrey	5 (2.2)	United Kingdom	University of Amsterdam	9 (2.6)	Netherlands
9th	Universiteit van Amsterdam	12 (1.8)	Netherlands	Bar-Ilan University	6 (3.4)	United States	University of Jyväskylä	5 (2.2)	Finland	University of Bedfordshire	9 (2.5)	United Kingdom
10th	University of Reading	12 (1.8)	United Kingdom	University of Haifa	5 (2.8)	Israel	University of Louisiana at Lafayette	5 (2.2)	United States	University of California, Los Angeles	9 (2.5)	United States

Further analysis found that only four institutions were listed in the rank lists throughout the three periods, including ETS, the University of California, the University of Melbourne, and the Lancaster University. Among these institutions, ETS, the Lancaster University and the University of Melbourne remained relatively steady in ranking order in terms of publication productivity, while the University of California, Los Angeles experienced a noticeable decline from being ranked 1st during the period 1984–1995 to being ranked 3rd during the period 1995–2006 and then to being ranked 10th during the period 2007–2008. The University of Reading (ranked 10th) contributed more publications during the first period; however, it produced a decreasing number of contributing publications during the second and third periods. In recent decades, certain institutions have contributed more publications and entered the rank list, e.g., the University of Illinois system, the University of California system, and the University of Georgia system, indicating that these institutions have attached a great deal of importance to language testing research and made fruitful achievements.

### The Most Frequently Collaborative Countries/Regions

Before reporting the most frequently collaborative countries/regions, we identified and calculated the total number of collaborative publications, international collaborative publications (publications including collaborators from different countries/regions) and collaborative countries/regions. The results showed that there were a total of 477 collaborative publications, accounting for 62.8% of the total number of publications, while there were only 188 international collaborative publications, accounting for 24.8% of the total. The results revealed that most researchers placed importance on collaborative publications, while less attention was given to international collaborative publications.

Table 8 presents countries/regions most frequently engaging in collaboration during the entire 37 years and during each of the periods. The results showed that the top 10 countries/regions contributed 402 collaborative publications and 143 international collaborative publications, accounting for 84.3 and 76.1% of the total number of publications in those categories, respectively. Among the top 10 contributing countries, America boasted the most collaborative publications (*N* = 174, 36.5%) and international collaborative publications (*N* = 41, 21.8%) and collaborated with the most countries (*N* = 18). The United Kingdom (ranked 2nd) and Australia (ranked 3rd) also produced a large number of collaborative publications (*N* = 59 and 51, respectively) and international collaborative publications (*N* = 31 and 21, respectively) and collaborated with the greatest number of countries/regions (*N* = 15 and 13, respectively) compared with the other seven countries/regions.

**TABLE 8:** Top 10 most frequently collaborative countries/regions.

																
Order	1984–2020				1984–1995				1989–2007				2008–2020			
	Countries/regions	Collaborative publications *N*/%	International collaborative publications *N*/%	Collaborative countries *N*	Countries/regions	Collaborative publications *N*%	International collaborative publications *N*/%	Collaborative countries *N*	Countries/regions	collaborative publications *N*/%	international collaborative publications *N*/%	collaborative countries *N*	Countries/regions	collaborative publications *N*%	international collaborative publications *N*/%	collaborative countries *N*
1st	United States	174/36.5	41/21.8	18	United States	37/52.3	6/42.9	5	United States	30/22.2	10/14.9	7	United States	107/39.3	25/23.3	14
2nd	United Kingdom	59/12.4	31/16.5	15	United Kingdom	11/15.7	3/21.4	1	United Kingdom	23/17.0	10/14.9	12	Australia	32/11.8	15/14.0	9
3rd	Australia	51/10.7	21/11.2	13	Israel	7/10.0	1/7.1	1	Australia	13/9.6	4/6.0	3	United Kingdom	25/9.2	18/16.8	9
4th	Canada	26/5.4	12/6.4	8	Australia	6/8.6	2/14.3	2	Canada	7/5.2	4/6.0	4	China	18/6.6	8/7.5	3
5th	Netherlands	24/5.0	4/21.3	7	Germany	2/2.9	0/0.0	0	China	5/3.7	3/4.5	2	Canada	18/6.6	7/6.5	5
6th	China	23/4.8	11/5.9	4	Netherlands	2/2.9	0/0.0	0	Netherlands	9/6.7	4/6.0	7	Japan	11/4.0	6/5.6	5
7th	Japan	15/3.1	7/3.7	5	Canada	1/1.4	1/7.1	1	Japan	4/3.0	1/1.5	1	Netherlands	13/4.8	0/0.0	0
8th	Israel	12/2.5	5/2.7	4	South Korean	1/1.4	1/7.1	1	South Korea	6/4.4	4/6.0	1	South Korea	4/1.5	3/2.8	2
9th	South Korea	11/2.3	8/4.3	2	Denmark	1/1.4	0/0.0	0	Israel	5/3.7	4/6.0	4	Germany	5/1.8	3/2.8	2
10th	Germany	7/1.5	3/1.6	9	France/Hungary	1/1.4	0/0.0	0	New Zealand	5/3.7	5/7.5	3	Turkey	4/1.5	0/0.0	0

Further analysis of the results across periods showed that the number of collaborative publications and international collaborative publications increased over time, specifically, from 69 and 14 publications, respectively, during the period 1984–1995 to 107 and 49 publications, respectively, during the period 1996–2007, and then to 237 and 85 publications, respectively, during the period 2008–2020. These results indicated that researchers in some countries/regions realized the significance of cooperation in research and have attached increasing importance to exchange and cooperation across institutions and countries. Moreover, the number of collaborating countries increased over the periods from 14 collaborative countries during the first period, to 29 countries during the second period and then to 53 countries during the third period.

We found that the United States, the United Kingdom, Australia, Netherlands, Canada, and South Korea remained in the rank list throughout the three periods. Moreover, these countries/regions that most frequently engaged in collaboration were also the most productive countries/regions, indicating that more emphasis on cooperation in research likely promotes greater publication productivity. In other words, the higher publication productivity in these countries was probably the result of more frequent collaboration within and across institutions and countries. For instance, the number of collaborative publications in China and Japan demonstrated a steady rise and allowed those countries to maintain their positions in the rank lists in recent decades; moreover, those countries also contributed to a high number of publications during those periods.

## Conclusion and Implications

This study employed a bibliometric analysis of publications in *Language Testing* since its establishment (from 1984 to 2020) to provide various types of bibliometric information about the journal to identify research trends and developmental patterns in language testing research. This bibliometric study discovered the following major findings with important implications.

First, apart from analyzing the common bibliometric information (e.g., most the frequently researched topics, and the most cited publications and authors), our study also included bibliometric information concerning test types by combining the journal’s aims and scopes and the interests of readers and researchers. This result is helpful in obtaining a more complete understanding of research trends and development patterns and providing guidance for future research. Thus, we suggest that future bibliometric studies expand the bibliometric data sources according to research aims and readers’ interests. In addition, we found that in the existing bibliometric research, the identification and analysis of research topics face great challenges in ensuring validity and reliability. For example, by adopting software (e.g., AntConc and CiteSpace) to analyze the research topics, this research can ensure higher reliability, but it may have lower validity because some of the analysis results were incapable of describing meaningful topics in the field. However, higher validity may be obtained by using manual analysis by multiple researchers concerning the title, abstract and contents of each publication, but lower reliability may result due to the subjectivity of topic analysis. Thus, future bibliometric research can consider enhancing the validity and reliability of research topic analysis by combining software and manual analysis.

Second, although different scales of tests were involved in the journal articles, regional and international large-scale high stakes language tests received greater amounts of attention from researchers than did classroom language tests/assessments. Moreover, there has been a sharp decrease in the research on classroom tests/assessments in recent years. It is understandable that regional and international tests would gain widespread attention due to their powerful influence within a country/region and even worldwide. Tests/assessments have multiple uses, e.g., selection, placement, evaluation and diagnosis. In most cases, classroom tests/assessments are used to diagnose strengths and weaknesses in teaching and learning so that they can provide helpful feedback for teaching and learning (Zou, 2011). Thus, researchers should pay more attention to classroom tests/assessments in the future and the journal should also increase its attention to classroom tests/assessments and even publish a special issue on classroom tests/assessments if necessary.

Third, through the analysis of the research topics, the most cited publications and the most cited authors (as measured by references), we found that some topics remained highly popular throughout all periods as well (e.g., validity/validation, reliability, test design and development, and vocabulary tests); however, several topics underwent a distinct decrease in level of interest (e.g., test method and test ethics), probably due to shifts in research foci. The topic with the highest level of interest is validity/validation. Research on these topics helps us gain a deeper and more thorough understanding of language testing issues. During the analysis of publications, we found that although the same topic was discussed across periods, different research methods were adopted during each period. For instance, regarding validity/validation, in the early period, test content information, test performance and think-aloud protocols were utilized to gain insight into the construct validity of reading tests (e.g., Anderson et al., 1991), whereas during recent periods, eye-tracking technology was used to explore the cognitive validity of reading tests (e.g., Bax, 2013). Thus, it is suggested that language testing research continue to improve the research methods and take full advantage of new scientific and technological approaches (e.g., functional near-infrared spectroscopy, FNIRS) in the development of and research into language testing and that the journal also keeps pace with the newest research developments. In addition, although some recent topics failed to make the top 10 list partly due to the age effect, they represent the latest developments in language testing research, such as research on cognitive diagnostics (e.g., Yi, 2016; Toprak and Cakir, 2020). These findings showed that language testing research incorporates theories and practices from other disciplines, e.g., cognitive psychology and motivational psychology, thereby expanding and enriching the directions of the discipline. Thus, it is hoped that language testing researchers continue to broaden their exploration of theories and practices from other disciplines and seek new interdisciplinary approaches to this research. To achieve these goals, language testing researchers should emphasize collaborations with scholars from other disciplines. Academic institutions and government agencies should also provide more funds and grants for interdisciplinary research.

Fourth, the publications were produced throughout a wide range of countries/regions and institutions, suggesting that the journal has provided a great venue to language testing researchers around the world who wish to publish their research. However, most contributing countries/regions and institutions have been located in developed countries in Asia, Europe and North America, while few were located in developing countries in Asia, Africa and Central/South America. It is important for *Language Testing* to expand the range of contributing countries/regions and increase the number of contributions from developing countries/regions to ensure an international scope for the publication, thereby promoting the achievement of its mission and aims. This study also found that collaborative publishing received more attention, while international collaborative publications received less attention. Fortunately, collaborative publications and international collaborative publications exhibited a significant increase in recent periods. Thus, researchers should attach more importance to and strengthen their focus on exchanges and cooperation, especially internationally, to broaden their research horizons, avoid duplication of research, and achieve superior research results.

This study also has a few limitations. Only the bibliometric data from *Language Testing* were used, which might not produce a complete picture of research trends and development patterns in language testing. Although we made every effort to improve the reliability of the research topic classification, the division of topics is still relatively subjective. In addition, due to space limitations, only the bibliometric data for the top ten examples of each category were reported, which is likely to provide limited understanding of research trends and developmental patterns in language testing to some extent. Thus, we should interpret the results of this study with caution.

## Data Availability Statement

The original contributions presented in the study are included in the article/supplementary material, further inquiries can be directed to the corresponding author.

## Author Contributions

CG, YZ, and RY collected and analyzed the data. All authors contributed to the article and approved the submitted version.

## Conflict of Interest

The authors declare that the research was conducted in the absence of any commercial or financial relationships that could be construed as a potential conflict of interest.

## Publisher’s Note

All claims expressed in this article are solely those of the authors and do not necessarily represent those of their affiliated organizations, or those of the publisher, the editors and the reviewers. Any product that may be evaluated in this article, or claim that may be made by its manufacturer, is not guaranteed or endorsed by the publisher.

## References

[B1] Allen D. (2016). Japanese cram schools and entrance exam washback. *Asian J. Appl. Linguist.* 3 54–67.

[B2] Anderson N. Bachman L. Perkins K. Cohen A. (1991). An exploratory study into theconstruct validity of a reading comprehension test: triangulation of data sources. *Lang. Test.* 8 41–46. 10.1177/026553229100800104

[B3] Bachman L. F. (1990). *Fundamental Considerations In Language Testing.* Oxford: Oxford University Press.

[B4] Bachman L. F. (1991). What does language testing have to offer? *TESOL Q.* 25 671–704. 10.2307/3587082

[B5] Bachman L. F. Palmer A. S. (1982). The construct validation of some components of communicative proficiency. *TESOL Q.* 16 449–465. 10.2307/3586464

[B6] Bachman L. F. Brian K. L. Mason M. (1995). Investigating variability in tasks and rater judgements in a performance test of foreign language speaking. *Lang. Test.* 12 238–257. 10.1177/026553229501200206

[B7] Bax S. (2013). The cognitive processing of candidates during reading tests: evidence from eye-tracking. *Lang. Test.* 30 441–465. 10.1177/0265532212473244

[B8] Beglar D. (2010). A rasch-based validation of the vocabulary size test. *Lang. Test.* 27 101–118. 10.1177/0265532209340194

[B9] Bosker H. R. Pinget A. F. Quene H. Sanders T. Jong N. (2013). What makes speech sound fluent? The contributions of pauses, speed and repairs. *Lang. Test.* 30 159–175. 10.1177/0265532212455394

[B10] Brown A. (1995). The effect of rater variables in the development of an occupation-specific language performance test. *Lang. Test.* 12 1–15. 10.1177/026553229501200101

[B11] Brown A. (2003). Interviewer variation and the co-construction of speaking proficiency. *Lang. Test.* 20 1–25. 10.1191/0265532203lt242oa

[B12] Butler Y. G. Lee J. (2010). The effects of self-assessment among young learners of English. *Lang. Test.* 27 5–31. 10.1177/0265532209346370

[B13] Cheng L. (2008). The key to success: english language testing in China. *Lang. Test.* 25 15–38. 10.1177/0265532207083743

[B14] Choi I. C. (2008). The impact of EFL testing on EFL education in Korea. *Lang. Test.* 25 39–62. 10.1177/0265532207083744

[B15] Cohen A. D. (1984). On taking language tests: what the students report. *Lang. Test.* 1 70–81. 10.1177/026553228400100106

[B16] Cumming A. (1990). Expertise in evaluating second language compositions. *Lang. Test.* 7 31–51. 10.1177/026553229000700104

[B17] Davies A. (1990). *Principles of Language Testing.* Oxford: Blackwell.

[B18] De Bellis N. (2009). *Bibliometrics and Citation Analysis: From the Science Citation Index to Cybermetics.* Lanham, MD: Scarecrow Press.

[B19] Dong M. X. Yin X. X. Luo Z. X. (2021). Research trend of business English in China: a bibliometric analysis. *Bus. English Res.* 1 28–38.

[B20] Douglas D. (1995). Developments in language testing. *Annu. Rev. Appl. Linguist.* 15 166–187. 10.1017/S0267190500002671

[B21] Eckes T. (2008). Rater types in writing performance assessments: a classification approach to rater variability. *Lang. Test.* 25 155–185. 10.1177/0265532207086780

[B22] Gingras Y. (2016). *Bibliometrics And Research Evaluation: Uses And Abuses.* Cambridge, MA: MIT Press.

[B23] Gong Y. Lyu B. Gao X. (2018). Research on teaching Chinese as a second or foreign language in and outside mainland China: a bibliometric analysis. *Asian Pacific Educ. Res.* 27 277–289.

[B24] Inbar-Lourie O. (2008). Constructing a language assessment knowledge base: a focus on language assessment courses. *Lang. Test.* 25 385–402. 10.1177/0265532208090158

[B25] Jiang J. (2018). Empirical studies on Foreign Language Testing in China: development trend and review and prospect. *Foreign Lang. World* 2 40–48.

[B26] Knoch U. (2009). Diagnostic assessment of writing: a comparison of two rating scales. *Lang. Test.* 26 275–304. 10.1177/0265532208101008

[B27] Kong J. F. (2017). The overview of use of Eye tracking technology in language assessment research. *Foreign Lang. Test. Teach.* 3 51–59, 64. 10.3791/57694 30582604

[B28] Kunnan A. J. (1998). *Validation in Language Assessment.* Mahwah, NJ: Lawrence Erlbaum Associates.

[B29] Language Testing Research Center (2021). Available online at:https://arts.unimelb.edu.au/language-testing-research-centre (Accessed August 28, 2021).

[B30] Language Testing (2021). *Journal Description.* Available online at: https://journals.sagepub.com/description/LTJ (Accessed August 28, 2021).

[B31] Laufer B. Nation P. (1999). A vocabulary-size test of controlled productive ability. *Lang. Test.* 16 33–51. 10.1177/026553229901600103

[B32] Lei L. Liu D. (2019a). The research trends and contributions of System’s publications over the past four decades (1973-2017): a bibliometric analysis. *System* 80 1–13. 10.1016/j.system.2018.10.003

[B33] Lei L. Liu D. (2019b). Research trends in applied linguistics from 2005-2016: a bibliometric analysis and its implications. *Appl. Linguist.* 40 540–561.

[B34] Leydesdorff L. Wagner C. (2009). Is the United States losing ground in science? A global perspective on the world science system. *Scientometrics* 78 23–36. 10.1007/s11192-008-1830-4

[B35] Liao S. Lei L. (2017). What we talk about when we talk about corpus: a bibliometric analysis of corpus-related research in linguistics (2000-2015). *Glottometrics* 38 1–20.

[B36] Liu X. Xu Q. Li M. (2015). A comparative analysis of scientific publications in management journals by authors from Mainland China, Hong Kong, Taiwan, and Macau: 2003-2012. *Scientometrics* 105 135–143.

[B37] Lumley T. (2002). Assessment criteria in a large-scale writing test: what do they really mean to the raters? *Lang. Test.* 19 246–276. 10.1191/0265532202lt230oa

[B38] Lumley T. Mcnamara T. F. (1995). Rater characteristics and rater bias: implications for training. *Lang. Test.* 12 54–71. 10.1177/026553229501200104

[B39] Malvern D. Richards B. (2002). Investigating accommodation in language proficiency interviews using a new measure of lexical diversity. *Lang. Test.* 19 85–104. 10.1191/0265532202lt221oa

[B40] Matsuno S. (2009). Self-, peer-, and teacher-assessments in Japanese university EFL writing classrooms. *Lang. Test.* 26 75–100. 10.1177/0265532208097337

[B41] McCarthy P. M. Jarvis S. (2007). Vocd: a theoretical and empirical evaluation. *Lang. Test.* 24 459–488. 10.1177/0265532207080767

[B42] Meara P. Buxton B. (1987). An alternative to multiple choice vocabulary tests. *Lang. Test.* 4 142–154. 10.1177/026553228700400202

[B43] Messick S. (1996). Validity and washback in language testing. *Lang. Test.* 13 241–256. 10.1177/026553229601300302

[B44] Moiwo J. Tao F. (2013). The changing dynamics in citation index publication position China in a race with the USA for global leadership. *Scientometrics* 95 1031–1050.

[B45] Osareh F. (1996). Bibliometncs, citation analysis and co-cication analysis: a review of literature I. *Libri* 46 149–158. 10.1515/libr.1996.46.3.149

[B46] Palmer A. Bachman L. F. (1981). “Basic concerns in test validation,” in *ELT Documents 111- Issues in Language Testing*, eds Alderson C. Hughes A. (London: The British Council), 135–151.

[B47] Phakiti A. (2003). A closer look at the relationship of cognitive and metacognitive strategy use to EFL reading achievement test performance. *Lang. Test.* 20 26–56. 10.1191/0265532203lt243oa

[B48] Pritchard A. (1969). Statistical bibliography or bibliometrics? *J. Doc.* 25 348–349.

[B49] Qi L. (2004). *The Intended Washback Effect Of The National Matriculation English Test In China:Intentions And Reality.* Beijing: Foreign Language Teaching and Research Press.

[B50] Qi L. (2007). Is testing an efficient agent for pedagogical change? Examining the intended washback of the writing task in a high-stakes English test in China. *Assess. Educ.* 14 51–74.

[B51] Read J. (1993). The development of a new measure of L2 vocabulary knowledge. *Lang. Test.* 10 355–371. 10.1177/026553229301000308

[B52] Ross S. (1998). Self-assessment in second language testing: a meta-analysis and analysis of experiential factors. *Lang. Test.* 15 1–20. 10.1177/026553229801500101

[B53] Schmitt N. Schmitt D. Clapham C. (2001). Developing and exploring the behaviour of two new versions of the Vocabulary Levels Test. *Lang. Test.* 18 55–88. 10.1177/026553220101800103

[B54] Shohamy E. (1984). Does the testing method make a difference? The case of reading comprehension. *Lang. Test.* 1 147–170. 10.1177/026553228400100203

[B55] Toprak T. Cakir A. (2020). Examining the L2 reading comprehension ability of adult ELLs: Developing a diagnostic test within the cognitive diagnostic assessment framework. *Lang. Test.* 38 106–131. 10.1177/0265532220941470

[B56] van Doorslaer L. Gambier Y. (2015). Measuring relationships in translation studies. On affiliations and keyword frequencies in the translation studies bibliography. *Perspectives* 23 305–319.

[B57] van Raan A. F. L. (2005). Fatal attraction: conceputal and methodological problems in the ranking of universities by bibliometric methods. *Scinetometrics* 62 133–143. 10.1007/s11192-005-0008-6

[B58] Wall D. Alderson J. (1993). Examining washback: the Sri Lankan Impact Study. *Lang. Test.* 10 41–69. 10.1177/026553229301000103

[B59] Weigle S. C. (1994). Effects of training on raters of ESL compositions. *Lang. Test.* 11 197–223. 10.1177/026553229401100206

[B60] Weigle S. C. (1998). Using FACETS to rater training effects. *Lang. Test.* 15 263–287. 10.1177/026553229801500205

[B61] White H. D. McCain K. W. (1989). Bibliometrics. *Annu. Rev. Inf. Sci. technol.* 24 119–186.

[B62] Wigglesworth G. Storch N. (2009). Pair versus individual writing: effects on fluency, complexity and accuracy. *Lang. Test.* 26 445–466. 10.1177/0265532209104670

[B63] Yang M. Z. (2002). The development of foreign language testing in the 1990s. *Foreign Lang. Teach.* 23 39–46.

[B64] Yi Y. (2016). Probing the relative importance of different attributes in L2 reading and listening comprehension items: an application of cognitive diagnostic models. *Lang. Test.* 34 337–355. 10.1177/0265532216646141

[B65] Zhang P. X. Fan J. S. Jia W. F. (2021). A bibliometric analysis of research hotspots and trends in international language testing (2008-2018). *Foreign Lang. Teach. Res.* 53 618–627, 641. 10.19923/j.cnki.fltr.2021.04.012

[B66] Zou S. (2011). *An Introduction to English Language Testing.* Beijing: Higher Education Press.

[B67] Zou S. Dong M. X. (2014). An overview of washback studies in China between 1994 and 2013. *Foreign Lang. China* 11 4–14.

